# The Prevalence and Factors Associated with Symptoms of Post-Traumatic Stress Disorder (ptsd) Among Adolescents Aged 15–18 in Western Cape, South Africa

**DOI:** 10.1007/s40653-026-00941-z

**Published:** 2026-07-06

**Authors:** Cebokazi Ngcakani-Mtati, Emily Garman, Chesney Ward-Smith, Katherine Sorsdahl, Claire Van Der Westhuizen

**Affiliations:** Department of Psychiatry; Centre for Public Mental Health, University of Cape Town, Rondebosch, South Africa

**Keywords:** Trauma exposure, Posttraumatic stress disorder (PTSD), Adolescents, Prevalence, Risk factors, South Africa

## Abstract

This study examined the prevalence and associated factors of post-traumatic stress disorder (PTSD) symptoms among 733 adolescents aged 15–18 years in low-resourced schools in the Western Cape, South Africa. Cross-sectional data from a 2022 survey were analysed using validated measures of mental health and psychosocial variables. Data were drawn from a school-based survey conducted in 2022 among 733 adolescents aged 15–18 years in the Western Cape Province of South Africa. Data were collected from 12 schools in collaboration with two nonprofit organisations that provide psychosocial services to learners in the community. Participants completed standardized measures assessing PTSD, depression, anxiety, and related demographic and psychosocial factors. A multiple linear regression model was used to examine associations between demographic and psychological factors and PTSD symptom severity. Most participants (92.6%) reported exposure to at least one traumatic event, with a mean of 4.7 events. The prevalence of clinically significant PTSD symptoms was 31%. Regression analyses identified female gender, anxiety, depression, bullying (victimisation and perpetration), and emotional regulation difficulties as significant predictors of higher PTSD symptoms. Socioeconomic status, social support, self-esteem, substance use, and physical activity were not significant. The findings highlight a high burden of trauma-related distress and the need for integrated adolescent mental health interventions.

## Introduction

Trauma exposure in sub-Saharan (SSA) is a significant issue, with a high proportion of adolescents experiencing traumatic events. A multi-country study conducted in 5 SSA countries found that among the 11 498 young people aged 18–24 years, 72–82% reported having experienced at least one form of traumatic event before the age of 18 years (Amene et al., 2024). In South African community samples, exposure to one or more traumatic events has also been reported at high levels of 77–92% (Closson et al., 2016; Hiscox et al., 2021; Seedat et al., 2000). A recent study conducted in Western Cape schools with 621 children aged 10–14, 81% reported having experienced bullying and 79% reported witnessing physical violence between adults in the home (Mkhize et al., 2024). Concerningly, literature suggests that around 16% of adolescents who have experienced a traumatic event go on to develop symptoms of Post Traumatic Stress Disorder (PTSD) (Alisic et al., 2014). Furthermore, research in low- and middle-income countries (LMICs) further suggests that around 28.5% of trauma-exposed adolescents endorse subthreshold PTSD symptoms, while approximately 6–15% meet the full Diagnostic and Statistical Manual of Mental Disorders, Fifth Edition (DSM-5) diagnostic criteria for PTSD (Stupar et al., 2021).

PTSD represents a significant mental health burden among children and adolescents in LMICs. A systematic review reported that the prevalence of PTSD among children and adolescents is substantially greater in low- and middle-income countries (LMICs) compared to high-income countries, with estimates varying from 0.2% to 87% (Benjet et al., 2016). A recent meta-analysis limited to studies conducted in SSA reported a pooled prevalence of 12.5% for PTSD among children and adolescents (Jakobsson et al., 2024). A previous meta-analysis reported that approximately 21.5% of adolescents between 10 and 19 years old in SSA were estimated to have PTSD (Jorns-Presentati et al., 2021). Although there is limited data on the prevalence of PTSD among adolescents in South Africa, given the frequency of traumatic exposures reported and the risk for developing PTSD after exposure, we anticipate a high prevalence of this condition. Available data from South Africa does indicate that PTSD is prevalent among adolescents. For example, a study of adolescents from low-socioeconomic communities in KwaZulu-Natal, South Africa found that 6% met the criteria for PTSD and an additional 4% reported clinically significant subthreshold symptoms, while a Western Cape-based study found that approximately 28% of the adolescents were experiencing subthreshold PTSD symptoms (Hiscox et al., 2021; Swain et al., 2017).

Multiple interrelated factors, ranging from the nature of the traumatic event to individual, household, family and community characteristics, significantly influence the development of PTSD symptoms in adolescents. First, characteristics of the traumatic event are crucial factors in determining the risk of developing PTSD (Al Jowf et al., 2022; Bouchard et al., 2023; Creamer et al., 2001; Golitaleb et al., 2022), with interpersonal violence, such as sexual assault and physical assault, being commonly associated with the development of PTSD (Seedat et al., 2004). Bullying has been associated with PTSD symptoms, as well as an increased risk of suicide attempts among the adolescents (Chen & Elklit, 2018; Idsoe et al., 2012). Further, the involvement of certain individuals in the traumatic event, such as family members or trusted figures, can significantly affect the victim’s psychological response. Trauma involving close family relationships, including family death and intrafamilial perpetration, is associated with greater post-traumatic stress symptoms and higher PTSD prevalence among children and adolescents (Alisic et al., 2014; Christoffersen & Thorup, 2024; Langeland & Olff, 2008; Mgoqi-Mbalo et al., 2017).

At the individual level, various factors have been associated with the risk of developing PTSD following trauma, while others may serve as protective factors. The literature highlights gender-specific risk, with females generally more prone to PTSD than males (Alisic et al., 2014; Hiscox et al., 2021; McLaughlin et al., 2013; Roberts et al., 2012; Tolin & Foa, 2006). Furthermore, research shows that adolescents with pre-existing mental health and substance use conditions are at increased risk compared to those without them (Copeland et al., 2007; Davis & Siegel, 2000; Hapke et al., 2006; McLaughlin et al., 2013). Adolescents with substance use conditions are approximately three times more likely to have experienced trauma and five times more likely to develop PTSD (Basedow et al., 2020). Additionally, studies investigating protective factors in this area show that physically active adolescents tend to experience better mental health post-trauma than inactive peers. A systematic review of 33 studies found that physical activity is positively linked to resilience and well-being, and negatively correlated with anxiety, depression, tension, and PTSD among adolescents (Wang et al., 2023). Further, studies found that personal traits such as self-esteem, intellectual functioning, resilience, and emotional and cognitive responses also decrease PTSD risk among adolescents (Breslau et al., 2006; Davis & Siegel, 2000; DiGangi et al., 2013; Dyregrov & Yule, 2006; Kataoka et al., 2012; McDonald & Deatrick, 2011; Pat-Horenczyk et al., 2013; Surzykiewicz et al., 2022; Vivrette et al., 2018; Willenberg et al., 2020).

Family and community contexts play an important role in increasing or mitigating PTSD risk. Poor family functioning and support can increase the risk of PTSD in adolescents (Kazak et al., 2006; Trickey et al., 2012) while social support and parental monitoring are linked to better outcomes (Stansfeld et al., 2017). Domestic violence within conflictual home settings may precipitate PTSD and dissociative symptoms among children and adolescents by exposing them to intimate partner violence and elevating the risk of physical and sexual victimisation (Christoffersen & Thorup, 2024; Cortina, 2012; Evans et al., 2008; Koolick et al., 2016; Levendosky et al., 2013; Olojede, 2018). Additionally, community characteristics, such as high levels of community violence and the normalization of violence, can increase adolescents’ risk of victimization and trauma exposure, and consequently contribute to the development of PTSD and other mental health conditions (Christoffersen & Thorup, 2024; Olojede, 2018; Stansfeld et al., 2017).

There is limited research on PTSD risk factors within the South African context, with most evidence available from high-income countries. However, risk factors in the South African context may differ due to unique socio-economic conditions, high levels of community violence, and structural inequalities present in the country. Given the limited published evidence on the prevalence of PTSD and factors associated with PTSD among South African adolescents, this study aims to contribute to the evidence base by examining demographic and psychological factors associated with PTSD symptoms among adolescents aged 15–18 years.

## Methods

### Study Design

This study utilized quantitative data from a school-based survey conducted between March and August 2022 among 733 adolescents aged 15–18 years in the Western Cape Province of South Africa. The dataset was collected as part of a larger study, the methods and procedures of which are summarized below and described in greater detail by Ward-Smith et al., (2024). Ethical approval for the original study was obtained from the Western Cape Education Department and the Human Research Ethics Committee within the Faculty of Health Sciences at the University of Cape Town. Ethical approval for the present study, which involved further analysis of the existing dataset to address new research questions, was obtained from the Human Research Ethics Committee at the University of Cape Town (HREC REF: 090/2025).

### Setting

The study was conducted in 12 high schools located in various low-resourced communities in the Western Cape, specifically the greater Cape Town area, the Winelands, and the Southern Peninsula. Of the 12 schools, 11 fall within the Western Cape Education Department’s quintile 1, 2, or 3 classifications, designated as no-fee or low-fee institutions.

### Sampling and Participants

In collaboration with Community Keepers (CK), Students for a Better Future (SBF), and Empilweni, the schools were identified considering geographic and language diversity. Written informed consent was obtained from participants aged 18 years. For participants younger than 18 years, written informed consent was obtained from a parent or primary caregiver, and written assent was obtained from the adolescent participant. Consent included permission for the data to be used in future analyses related to the study objectives. All participants recruited in the original survey were eligible for inclusion in the present study.

### Procedures

The study team engaged with the school principals and NGO liaisons regarding the study’s objectives, inclusion and exclusion criteria, and logistical details. Participant recruitment was conducted over a six-month period through participating schools. The NGO liaison was provided with printed materials, including a study brief, information sheets, and consent/assent forms, for distribution. NGO liaisons introduced the study to one class per grade (Grades 10–12), explained the process, and distributed the consent/assent forms for return within 1–2 weeks.

Following this period, the research team returned to conduct the survey with consenting/assenting learners at pre-arranged times. Prior to data collection, the researchers introduced themselves, led a brief breathing exercise, and provided a recap of the study. Participants were invited to ask questions before beginning the survey. The tablet-based survey was self-administered individually in participants’ preferred language (English, isiXhosa, or Afrikaans) and took approximately 45–60 min to complete. Participants with clinically significant scores on mental health measures received a pop-up notification upon completion of the survey, offering follow-up support with a clinical psychologist or social worker for further mental health screening and potential referral for additional psychological services.

### Measures

Participants completed self-administered questionnaires in English, measuring PTSD (CATS), depression (CES-D-10), anxiety (GAD-7), emotional regulation (DERS-16), self-esteem (RSES), bullying (BCS-A), substance use (ASSIST-Y), physical activity (PAQ-A), social support (MSPSS), and socioeconomic status.

#### PTSD

The presence of PTSD symptoms was measured using the Child and Adolescent Trauma Survey (CATS). The CATS is a self-report instrument assessing PTSD-qualifying stressors, other life events, and PTSD symptomatology (March, 1999). CATS items are scored on a 0–3 Likert scale, where 0 indicates ‘Never’ and 3 indicates ‘Almost always’. The tool has demonstrated strong internal consistency and test-retest reliability in international samples (Sachser et al., 2017). For this study, a cut-off score of ≥ 15 was used to identify clinically significant PTSD symptoms, based on Suliman et al., (2005), whose work validated this threshold in South African adolescents.

#### Depression

Risk for depression was evaluated using the Center for Epidemiological Studies Depression Scale (CES-D-10) (Bradley et al., 2010). The tool is a 10-item Likert scale questionnaire that measures depressive symptoms experienced over the past week, with scores ranging from 0 to 30. The CES-D-10 has been validated in several sub-Saharan African countries (Kilburn et al., 2018), and among a population including adults and adolescents (15–19 years) in South Africa, demonstrating strong psychometric properties (Baron et al., 2017). A cut-off score of ≥ 12 indicating depression was used on the CES-D-10, as suggested by Baron et al. (2017).

#### Anxiety

Risk of anxiety was assessed using the GAD-7 (Mossman et al., 2017). A cut off score of ≥ 10 was used on the GAD-7 (Ward-Smith et al., 2024). The responses are scored from 0 (not at all) to 3 (nearly every day) resulting in a total score ranging from 0 to 21. The GAD-7 has good construct validity (Rodriguez et al., 2025). The GAD-7 has been found appropriate to use across diverse populations and linguistic groups in South Africa (Rodriguez et al., 2025), and has been validated specifically among Xhosa-speaking adolescents in South Africa, demonstrating moderate criterion validity and strong construct validity (Marlow et al., 2023).

#### Bullying

The Bullying and Cyberbullying Scale for Adolescents (BCS-A) was utilized to evaluate face-to-face and cyberbullying incidents over the past three months. The BCS-A is a brief 26 item self-report instrument and consists of 2 sets of 13 items, addressing both traditional (physical or verbal aggression) and digital bullying, including victimization and perpetration, among adolescents (Thomas et al., 2019). Each item is rated on a 5-point Likert scale (0–4), where 0 is ‘never’ and 4 is ‘several times a week’. Higher scores indicate greater frequency of bullying involvement. Although there isn’t a published study that has validated the BCS-A specifically in South Africa, it has been shown to have strong psychometric properties in international studies showing acceptable concurrent, convergent and predictive validity over 6 months (Thomas et al., 2019).

#### Self-esteem

The Rosenberg Self-Esteem Scale (RSES) was used to assess participants’ self-esteem. The RSES is a brief, 10-item measure, five items of which are positively and five negatively worded. The scores range from 0 to 30, and the higher the total score, the higher the self-esteem. The RSES is the benchmark for assessing self-esteem and is known for its excellent reliability(Monteiro et al., 2021). The scale has been validated for use with the South African population demonstrating sound psychometric properties across diverse South African adult groups (Westaway et al., 2013), however its validity for use among adolescents has yet to be formally established.

#### Emotional regulation

Emotional regulation was assessed using the self-reported Difficulties in Emotion Regulation Scale 16 (DERS-16) (Bjureberg et al., 2016). This scale evaluates adaptive emotional responses, including (a) awareness, comprehension, and acceptance of emotions; (b) the ability to control behaviours during negative emotional states; (c) the flexible application of appropriate strategies to manage the intensity or duration of emotional reactions; and (d) openness to experiencing negative emotions. Participants rate each of the 16 items on a 5-point Likert scale ranging from 1 (almost never) to 5 (almost always). Higher scores indicate greater difficulties in emotional regulation (Bjureberg et al., 2016), and although a study has found that a cut-off of ≥ 34 may indicate emotional dysregulation among adults (Burton et al., 2022), local cutoffs for adolescents are not available. While the DERS-16 has demonstrated excellent internal consistency, reliability, and validity in international contexts (Bjureberg et al., 2016; Skutch et al., 2019; Yiğit & Guzey Yiğit, 2019), it has not yet been validated for use with South African adolescents.

#### Physical activity

Physical activity levels among participants were measured using the Physical Activity Questionnaire for Adolescents (PAQ-A) (Kowalski et al., 2004). The PAQ-A is an 8 item, self-administered instrument assessing typical engagement in moderate to vigorous physical activity in adolescents aged 14–20. Each item was rated on a 5-point Likert scale, with scores ranging from 1 (indicating low physical activity) to 5 (indicating high physical activity). Scores were binarized into low and moderate/high activity, using a cut-off point, where scores < 2.75 indicate low activity, and ≥ 2.75 indicate moderate to high activity (Kowalski et al., 2004). The PAQ-A has shown good internal reliability and acceptable construct validity in previous studies (Janz et al., 2008; Kowalski et al., 2004) but has yet to be validated for use among South African adolescents.

#### Substance use

Substance use was assessed using the self-rated Alcohol, Smoking and Substance Involvement Screening Test–Youth version (ASSIST-Y), a WHO-developed screening tool designed to identify risk related to alcohol, tobacco, and other substance use. (Humeniuk et al., 2016). The standard ASSIST contains eight items for each substance category and uses substance-specific weighted scoring, meaning items do not contribute equally to risk levels. Cut-off scores also vary by substance rather than applying a single threshold. The cutoff scores recommended by Humeniuk et al. (2016) were used in this study. While the tool has demonstrated strong internal reliability in studies with at-risk adolescents in Zambia (Kane et al., 2016), evidence from South Africa is limited to studies that employed the adult ASSIST rather than the youth version (van der Westhuizen et al., 2016).

#### Social support

To measure social support, the Multidimensional Scale of Perceived Social Support (MSPSS) was used (Zimet et al., 1988). This 12-item instrument evaluates perceived levels of social support from three sources: family, friends, and significant others. Each item is rated on a seven-point Likert scale, ranging from 1 (very strongly disagree) to 7 (very strongly agree). The total score, ranging from 12 to 84, is obtained by summing all items, with higher scores indicating greater perceived social support. The MSPSS has been extensively used and has demonstrated good internal consistency and construct validity for use among adolescents in South Africa (Bruwer et al., 2008).

#### Household poverty index

Participants’ access to basic daily necessities was assessed using the Household Index for Children and Adolescents in South Africa (Barnes & Wright, 2012), as a proxy for household poverty. This measure includes 8 items such as access to three meals per day, medical care, medication, adequate clothing, hygiene products (e.g., soap), school fees, a school uniform, and shoes. Responses were reverse scored (0 = access; 1 = no access), and the items were summed to generate a total poverty score, reflecting the number of unmet basic needs (thus, greater scores meaning greater poverty). Household poverty is categorized into low (0–2), moderate (3–5), and high poverty (6–8) based on the number of unmet basic needs.

### Data Analysis

Data were analysed using SPSS v29. Descriptive statistics, chi-square tests, and linear regression analyses were performed. Variables with *p* < .200 in unadjusted models were included in adjusted models, with age retained as a covariate. Results were reported as β coefficients with 95% confidence intervals.

## Results

### Characteristics of the Sample

The sociodemographic characteristics of the sample are described in Table 1. The sample consisted of 733 adolescents (*N* = 733) with a mean age of 16.8 years old (*SD* = 0.84). Most participants were females (*n* = 532, 72.6%), with a small proportion identifying as transgender (*n* = 10, 1.4%). Transgender participants (*n* = 10) were excluded from the analysis to ensure sufficient statistical power. Most participants reported living in households of 5 or more individuals (53%). With regards to financial circumstances, most households (*n* = 603, 82.3%) had at least one employed caregiver, with 17.7% (*n* = 130) reporting no employment. Most caregivers were employed full-time (*n* = 480; 65.5%), and nearly one-third (28.6%) lived in households with moderate to high poverty.

**Table 1:** Socio-demographic characteristics of the sample

	Total *n* = 733)
	n (%)
Gender	
Female	532 (72.6)
Male	191 (26.1)
Transgender	10 (1.4)
Age (m, sd)	**16.8 (0.84)**
15 years	66 (9.0)
16 years	169 (23.1)
17 years	373 (50.9)
18 years	125 (17.1)
Household number	
1 person	10 (1.4)
2 people	50 (6.9)
3 people	116 (16.0)
4 people	165 (22.7)
5 people	188 (25.9)
6 or more people	197 (27.0)
Caregiver employment	
Yes employed	603 (82.3)
Unemployed	130 (17.7)
Caregiver type of work	
None	64 (8.7)
Casual	66 (9.0)
Part-time	123 (16.8)
Full-time	480 (65.5)
Household poverty	
Low poverty (0–2)	524 (71.5)
Moderate poverty (3–5)	187 (25.6)
High poverty (6–8)	22 (3.0)

### Trauma exposure

Table 2 provides a breakdown of the different trauma types experienced by the participants in the whole sample and by gender, as measured by the CATS scale.

**Table 2:** Frequency of trauma exposure

Variables	Total (*n* = 723)	Female (*n* = 532)	Male (*n* = 191)	*p*-value	Pearson Chi-Square
	*n* (%)	*n* / (%)	*n* / (%)	*p* < .05	
Any trauma exposure				0.328	2.23
Yes	677 (92.6)	494 (93.2)	173 (90.6)		
No	54 (7.4)	36 (6.8)	18 (9.4)		
Number of traumatic events (m, sd)	4.7 (2.87)				
Humanitarian occurrences				0.619	0.96
No	489 (66.7)	356 (66.9)	125 (65.4)		
Yes	243 (33.2)	175 (32.9)	66 (34.6)		
Experience of interpersonal violence				0.444	1.62
No	266 (36.3)	198 (37.2)	66 (34.6)		
Yes	466 (63.7)	333 (62.6)	125 (65.4)		
Witnessing interpersonal violence				0.353	2.09
No	119 (16.3)	86 (16.2)	33 (17.3)		
Yes	612 (83.7)	444 (83.8)	158 (82.7)		
Sexual trauma				< 001	24.11
No	586 (79.9)	407 (76,6)	174 (91.1)		
Yes	146 (19.9)	124 (23.4)	17 (8.9)		
Sudden loss / severe illness				0.218	2.27
No	412 (56.2)	289 (54.4)	116 (60.7)		
Yes	320 (43.7)	242 (45.6)	75 (39.3)		
Serious accident/ injury (unintentional)/ medical procedure				0.043	3.87
No	358 (48.9)	272 (51.2)	82 (42.9)		
Yes	374 (51.1)	259 (48.8)	109 (57.1)		

Data analysis showed that most participants (*n* = 677; 92.6%) reported trauma exposure, with a mean of 4.7 events (SD = 2.87). The most common exposures were witnessing (83.7%) and experiencing (63.7%) interpersonal violence. Sexual trauma was the least reported category, though still experienced by 19.9% of the sample.

An investigation of gender differences shows that there was a statistically significant association between gender and exposure to sexual trauma, with females (23.4%) reporting more exposure to sexual trauma than males (8.9%; χ^2^ = 24.11; *p* < .001). In addition, there was a statistically significant association between gender and exposure to serious accidents or injury and medical procedures, with males (57.1%) reporting more exposure than females (48.8%; χ^2^ = 3.87; *p* < .043).

### Psychosocial Characteristics

Mental health related characteristics of the participants are presented in Table 3. Clinically significant PTSD symptoms were present in 31% of participants (M = 24.58; SD = 13.30). Over half screened positive for depression (51.7%) and anxiety (56.4%). Emotional regulation difficulties were moderate (M = 43.47; SD = 15.03), and 43.2% reported low self-esteem (M = 16.49; SD = 5.35). Most participants (83.7%) reported adequate social support. Bullying victimisation (M = 2.14; SD = 2.01), was more common than perpetration (M = 1.06; SD = 1.49). Physical activity was low (M = 2.30; SD = 0.71), and substance use risk was notable, particularly for alcohol (36%) and tobacco (33.4%).

**Table 3:** Psychosocial characteristics

Variable	Total	Female	Male
	*n* = 723 (%)	*n* = 532 (%)	*n* = 191 (%)
Symptoms of PTSD (CATS) (m, sd)	24.58 (13.30)		
No	497 (69.0)	346 (65.3)	151 (79.5)
Yes	223 (31.0)	184 (34.7)	39 (20.5)
Symptoms of depression (CESD) (m, sd)	**13.09 (5.79)**		
No	310 (42.9)	195 (36.7)	115 (60.2)
Yes	413 (57.1)	337 (63.3)	76 (39.8)
Symptoms of anxiety (GAD-7) (m, sd)	**10.76 (5.34)**		
No (0–9)	315 (43.6)	201 (37.8%)	114 (59.7)
Yes (10–21)	408 (56.4)	331 (62.2%)	77 (40.3)
Emotional Regulation (DERS) (m, sd)	**43.47 (15.03)**		
Self Esteem (RSES)	**16.49 (5.35)**		
Low	309 (43.2)	246 (46.7)	63 (33.5)
Moderate – high	406 (56.8)	281 (53.3)	125 (66.5)
Perceived Social Support (MSPSS) (m, sd)	**54.20 (17.52)**		
Low	119 (16.3)	96 (18.1)	22 (11.5)
Moderate – high	611 (83.7)	433 (81.9)	169 (88.5)
Bullying - Perpetrator (BCS-A) (m, sd)	**1.06 (1.49)**		
No	684 (93.3)	502 (94.4)	172 (90.1)
Yes	49 (6.7)	30 (5.6)	19 (9.9)
Bullying - Victim (BCS-A) (m, sd)	**2.14 (2.01)**		
No	607 (82.8)	442 (83.1)	156 (81.7)
Yes	126 (17.2)	90 (16.9)	35 (18.3)
Physical Activity (PAQ-A) (m, sd)	**2.30 (0.71)**		
Low physical activity	565 (78.9)	449 (85.2)	116 (61.4)
Moderate - High physical activity	151 (21.1)	78 (14.8)	73 (38.6)
Tobacco risky use			
No	481 (66.6)	356 (67)	125 (65.4)
Yes	241 (33.4)	175 (33)	66 (34.6)
Alcohol risky use			
No	462 (64.0)	325 (61.2)	137 (71.7)
Yes	260 (36.0)	206 (38.8)	54 (28.3)
Cannabis risky use			
No	593 (82.1)	447 (84.2)	146 (76.4)
Yes	129 (17.9)	84 (15.8)	45 (23.6)
Illicit drug risky use (excl. cannabis)			
No	638 (88.4)	460 (86.6)	178 (93.2)
Yes	84 (11.6)	71 (13.4)	13 (6.8)

### Factors Associated with PTSD Symptoms

We used linear regression to assess unadjusted and adjusted associations of sociodemographic variables (age, gender, household SES), risk (anxiety, depression, bullying, and substance use), and protective factors (self-esteem, emotional regulation, social support, and physical activity) (independent variables), with PTSD symptoms (dependent variable). Results of the unadjusted and adjusted models are presented in Table 4. All risk and protective factors, other than caregiver employment and age, were significant in the unadjusted models. Caregiver employment was excluded in the adjusted model.

**Table 4:** Unadjusted and adjusted associations between sociodemographic, lifestyle and psychosocial factors and PTSD

	Unadjusted		Adjusted	
Variable	β (CI)	*p* (< 0.05)	𝛽 (CI)	*p* (< 0.05)
Age	–0.09 (-1.29; 1.12)	0.890	0.22 (-0.62; 1.07)	0.608
Gender (n, %)	-6.75 (-8.98; -4.51)	< 0.001	-2.18 (-3.85; -0.52)	0.010
Caregiver employment (n, %)	0.08 (-2.71; 2.60)	0.954		
Household poverty	1.49 (0.93; 2.06)	< 0.001	0.25 (-0.18; -0.67)	-0.255
Symptoms of anxiety	1.65 (1.51; 1.79)	< 0.001	0.74 (0.54; 0.94)	< 0.001
Symptoms of depression	1.43 (1.30; 1.57)	< 0.001	0.34 (0.15; 0.53)	< 0001
Being bullied - victimisation	2.46 (2.00; 2.92)	< 0.001	0.88 (0.49; 1.27)	< 0.001
Bullying- perpetration	1.66 (1.01; 2.31)	< 0.001	0.56 (0.05; 1.06)	0.030
Total self-esteem	-1.09 (-1.26; -0.92)	< 0.001	-0.13 (-0.29; 0.03)	0.106
Emotional regulation	0.57 (0.52–0,62)	< 0.001	0.21 (0.15; 0.28)	< 0.001
Total social support	-0.14 (-0.19; -0.08)	< 0.001	0.03 (-0.01; 0.07)	0.174
Physical activity	-0.13 (-0.31; 0.04)	0.147	0.09 (-0.37; 0.22)	0.162
Tobal tobacco	0.49 (0.31; 0.67)	< 0.001	0.14 (-0.01; 0.30)	0.062
Total alcohol	0.48 (0.34; 0.62)	< 0.001	0.08 (-0.04; 0.20)	0.212
Total cannabis	0.37 (0.20; 0.54)	< 0.001	0.03 (-0.11; 0.16)	0.708
Total illicit drugs (w/o cannabis)	0.49 (0.32; 0.67)	< 0.001	0.07 (-0.07; 0.21)	0.321

Regression analyses showed that female gender, anxiety, depression, bullying (victimisation and perpetration), and emotional regulation difficulties were significantly associated with higher PTSD symptom severity. First, males reported significantly lower PTSD symptoms than females (β = -2.18, 95% CI: [-3.85 to -0.52], *p* = .010). Second, higher levels of anxiety symptoms (β = 0.74, 95% CI [0.54, 0.94], *p* < .001) were significantly associated with increased severity of PTSD symptoms. Third, greater depressive symptoms (β = 0.34, 95% CI [0.15, 0.53], *p* < .001) were positively associated with PTSD symptoms. Fourth, experiences of being bullied (victimization) increased the likelihood of experiencing PTSD symptoms (𝛽 = 0.88, 95% CI [0.49 to 1.27], *p* < .001), as did bullying perpetration (β = 0.56, 95% CI [0.05 to 1.06], *p* = .030). Lastly, greater emotional regulation difficulties were associated with greater PTSD symptoms (β = 0.21, 95% CI [0.15, 0.28], *p* < .001). Socioeconomic status (SES), social support, self-esteem, physical activity and substance use were not significantly associated with PTSD symptoms after adjusting for other variables in the model.

## Discussion

The study aimed to examine the prevalence and factors associated with symptoms of PTSD among adolescents aged 15–18 in the Western Cape. Three key findings emerged: (1) most adolescents reported exposure to at least one traumatic event, and gender differences emerged in two different types of traumatic exposures; (2) among the sample, the prevalence of PTSD symptoms was high; and (3) several factors were independently associated with greater PTSD symptom severity. In contrast, socioeconomic status, social support, self-esteem, physical activity, and substance use were not significantly associated with PTSD symptoms after adjustment for other variables.

First, the findings demonstrate that trauma exposure is prevalent among this population, with 92.6% of participants reporting exposure to at least one traumatic event. Among the different categories of trauma reported by adolescents in our sample, witnessing interpersonal violence (83.7%) and direct experiences of interpersonal violence (63.7%) were the most common. These findings are consistent with evidence from other SSA contexts, where an estimated 67–98% of children and adolescents are exposed to interpersonal violence, either as victims or witnesses (Hiscox et al., 2021; Kaminer et al., 2013; Seedat et al., 2004). This high prevalence reflects the pervasive nature of violence within many communities, resulting in frequent exposure to aggression and violence in both household and community settings (Leoschut & Kafaar, 2017). Moreover, the indirect nature of witnessing a traumatic event may influence how the event is remembered and experienced internally (helplessness) by the individual, which can shape the risk and severity of PTSD symptoms (Atwoli et al., 2013).

Research suggests that trauma type and PTSD risk can differ by gender (Alisic et al., 2014; Hiscox et al., 2021; McLaughlin et al., 2013; Roberts et al., 2012; Tolin & Foa, 2006). In the current study, there were few gender differences in type of traumatic exposures experienced, suggesting that trauma is widespread and broadly experienced regardless of gender. That said, females reported greater exposure to sexual trauma than males, whereas males reported greater exposure to serious accidents, injuries, and medical procedures, aligning with previous findings (McLaughlin et al., 2013; Seedat et al., 2004; Tolin & Foa, 2006; Williams et al., 2007). Beyond increasing PTSD risk, adolescent sexual assault is linked to poorer school performance, risky sexual behaviour, unplanned pregnancy, and relationship difficulties (Ackard & Neumark-Sztainer, 2002; Freeman & Temple, 2010).

Second, one-third of the sample scored above the clinical threshold for PTSD symptoms (31%), a prevalence that is slightly higher than those reported in other studies (Alisic et al., 2014; Hiscox et al., 2021; Seedat et al., 2004; Swain et al., 2017). To date, the only published South African validation of the CATS is that of Suliman et al. (2005), who compared the measure against a structured clinical interview in a Cape Town adolescent sample and identified an optimal cut-off score of 15. The present study therefore applied this locally derived threshold, whereas many international studies use a higher threshold (≥ 21). The use of a lower cut-off may increase sensitivity and reduce the likelihood of missing clinically significant cases, but it may also elevate prevalence estimates relative to studies applying more stringent thresholds.

Despite these considerations, the number of adolescents who screened above the clinical threshold is still significant from a clinical perspective. Adolescents with PTSD often experience significant impairments in adaptive, social, emotional, and academic functioning. These difficulties help explain many of the common challenges seen in traumatized youth and can affect not only their current well-being but also their long-term life trajectory (Vasilevski & Tucker, 2016). These findings highlight the immediate and long-term challenges facing many young people in South Africa.

Third, in the adjusted model, gender, anxiety, depression, bullying (victimization and perpetration), and emotional regulation difficulties remained significantly associated with symptoms of PTSD. Gender was significantly associated with PTSD symptoms, which aligns with literature demonstrating gender differences in trauma responses (Alisic et al., 2014; McLaughlin et al., 2013). Females had significantly higher PTSD, and this is consistent with previous studies, which found that women and girls show higher PTSD risk than boys across study contexts (Alisic et al., 2014; Hiscox et al., 2021; McLaughlin et al., 2013; Tolin & Foa, 2006). For example, a study conducted in a sample of adolescents from an impoverished community in South Africa reported that females have a significantly higher prevalence of PTSD compared to males, even when adjusting for trauma exposure and co-occurring depressive symptoms (Hiscox et al., 2021).

Depression and anxiety were both positively associated with PTSD, suggesting that participants with higher levels of depression and anxiety also tended to report more severe PTSD symptoms. This supports literature indicating that depression and anxiety are commonly comorbid with PTSD (Kar, 2009; Kessler et al., 2005; Kmett Danielson et al., 2010). From a diagnostic perspective, the DSM-5 PTSD criteria includes symptoms that overlap with mood and anxiety symptoms (APA, 2022), namely internalising symptoms such as persistent sadness, guilt and worry; this overlap may partially explain the associations found in this study. Experiences of bullying (victimization and perpetration) were also significantly associated with higher PTSD symptoms, suggesting that both victims and perpetrators may experience elevated trauma-related distress. Many studies have found an association between bullying victimization and PTSD (Idsoe et al., 2012; Li et al., 2023; Ossa et al., 2019); however, the association between bullying perpetration and PTSD is less commonly studied. A recent study among adolescents found that bullying perpetrators, particularly those who engaged in verbal offenses and physical aggression, reported elevated PTSD symptoms (Fernández-Aldana et al., 2024). This highlights the complex role of peer relations and adolescents’ social environments in maintaining or exacerbating trauma symptoms (Idsoe et al., 2020).

In this study, difficulties with emotional regulation significantly predicted PTSD symptoms, suggesting that that greater emotional dysregulation is independently linked to PTSD symptom severity. This is not surprising as emotional dysregulation is one of the core features of PTSD among children and adolescents, as described in the DSM-5 (APA, 2022). Adolescents with PTSD often display irritable behaviour, anger outbursts, and persistent negative emotional state, react with agitation or disorganized behaviour (APA, 2022; Dyregrov & Yule, 2006), and are more likely to exhibit increased impulsiveness (Astitene et al., 2018), self-harm and or suicidal behaviours (Cook et al., 2005). Further, the current study’s finding is consistent with literature showing that individuals experiencing symptoms of trauma often struggle with emotional regulation (Conti et al., 2023), and that there is a link between unhelpful emotional coping strategies and negative thinking patterns in individuals with PTSD (Ouhmad et al., 2023).

Several factors were not significantly associated with PTSD symptoms in the present study. The lack of association between SES and PTSD may reflect the relatively limited variability in socioeconomic circumstances within the sample, which was predominantly drawn from low socioeconomic communities. Similarly, social support and self-esteem were significantly associated with PTSD symptoms in unadjusted analyses but were no longer significant in the adjusted model, suggesting that these associations were accounted for by other correlated variables included in the model. Physical activity and substance use variables (tobacco, alcohol, cannabis, and other illicit drugs) were not independently associated with PTSD symptoms after adjustment. Overall, these findings suggest that in this sample, PTSD symptoms were more strongly associated with psychological and behavioural correlates than with broader psychosocial or lifestyle factors. Further research is needed to better understand protective processes and resilience following trauma exposure among South African adolescents.

The study’s findings must be considered in light of several limitations. First, the data were collected as part of a broader study investigating associations between depression, anxiety symptoms, and emotion regulation. As a result, the available measures may not have fully captured the breadth and complexity of PTSD and its associated risk factors. Second, the findings may not be generalizable to adolescents from more socioeconomically diverse backgrounds because the study sample was drawn from a relatively homogeneous low socioeconomic population, limiting the extent to which the findings can be assumed to apply across different socioeconomic contexts. Another limitation of the study is the uneven gender distribution, since the study included more female participants (72.6%) than males (26.1%). Additionally, while 10 transgender participants were included in the sample, their data were not analysed separately due to the small number, which limited the ability to draw meaningful or generalizable conclusions about this group. Third, self-administered questionnaires led to some missing data, and reliance on self-report may have introduced social desirability bias. (Ouhmad et al., 2023). Although the present study applied a South African cut-off score of ≥ 15 derived from the only published local validation study of the CATS among adolescents (Suliman et al., 2005), a further limitation is that the measure has undergone very limited psychometric validation in South African adolescent populations. While the use of a locally validated threshold strengthens the contextual relevance of the prevalence estimate, prevalence rates may differ from those reported in studies using alternative international cut-off scores. Therefore, although the study offers useful insight into PTSD symptoms among adolescents in low socioeconomic settings, the findings should be interpreted cautiously.

Despite these limitations, the results of the study carry meaningful implications for policy, practice, and future research. The relatively high proportion of adolescents scoring above the threshold for clinically significant PTSD symptoms (31%) highlights the need for early identification and intervention for trauma-related difficulties among adolescents to reduce the burden on adolescents, families, communities, and the broader social and economic landscape (Golberstein et al., 2019). Although there is a clear need for brief, accessible and contextually appropriate PSTD interventions, access to adequate mental health care, especially through primary healthcare, is still very limited in South Africa (Sorsdahl et al., 2023). South Africa’s Mental Health Policy & Strategic Plan (2023–2030) advocates for task-sharing mental health interventions to address the large treatment gap (Department of Health, 2023). However, very few studies have reported on trauma-focused interventions for adolescents that utilise task-sharing approaches. Given the association identified in the study between PTSD and elevated symptoms of depression and anxiety, as well as with emotional regulation difficulties, accessible interventions for adolescents in context that address these challenges may help reduce PTSD symptoms and improve overall wellbeing. Lastly, at present no additional peer-reviewed South African or SSA validation studies specifically evaluating the CATS tool beyond Suliman et al. (2005) have been published. Therefore, future research should focus on further validating the CATS and examining the appropriateness and diagnostic accuracy of PTSD cut-off scores across diverse South African contexts.

## Data Availability

The study used data from a 2022 school-based survey conducted by C. Ward-Smith (CWS) at the Centre for Public Mental Health. These data are not publicly available due to participant confidentiality but can be requested from the Centre for Public Mental Health with appropriate permissions.
